# Predicting Prostate Cancer Risk and Its Associated Factors Using Machine Learning Techniques: A Retrospective Study

**DOI:** 10.1002/hsr2.72052

**Published:** 2026-05-03

**Authors:** Serveh Mohammadi, Behzad Imani, Soheila Saeedi, Mohammad Ali Amirzargar

**Affiliations:** ^1^ Department of Operating Room, Mahabad School of Nursing Urmia University of Medical Sciences Urmia Iran; ^2^ Department of Operating Room, School of Paramedicine Hamadan University of Medical Sciences Hamadan Iran; ^3^ Department of Health Information Technology, School of Allied Medical Sciences Hamadan University of Medical Sciences Hamadan Iran; ^4^ Department of Urology, Faculty of Medicine Hamadan University of Medical Sciences Hamadan Iran

**Keywords:** artificial intelligence, machine learning algorithms, prostatic neoplasms, risk factors

## Abstract

**Background and Aims:**

Prostate cancer affects many people around the world and is fatal. Today, artificial intelligence‐based techniques are widely used to predict and diagnose diseases. This study aimed to use machine learning‐based techniques to predict and identify the most significant risk factors of prostate cancer.

**Methods:**

This retrospective study was conducted at the Shahid Beheshti Hospital, Hamadan Province, Iran, in five steps, including the identification of risk factors, data collection, preprocessing, modeling, and evaluation. The Web of Science, Scopus, Medline, and PubMed Central databases were searched from the beginning of 2000 to 2024. Different prediction algorithms, including Logistic Regression, Gradient Boosting, Random Forest, XGBoost, Support Vector Machine, and Neural Networks algorithms developed based on 49 confirmed factors. 597 medical records were reviewed. 342 patients (57.29%) had prostate cancer, and 255 patients (42.71%) had benign prostate hyperplasia. The algorithm's performance was evaluated using accuracy, precision, recall, F1‐score, and support metrics.

**Results:**

The results of this study showed the XG Boosting model performed best in predicting the effective factors, with an accuracy of 77.5% a sensitivity of 74.5%, and a specificity of 79.7%. Key predictors included total and free prostate‐specific antigen (PSA) levels, hemoglobin, BMI, and fish consumption.

**Conclusion:**

Machine learning can enhance and personalize the identification of potential risks of PCa, but more research is necessary to refine these algorithms and address data biases.

## 1 Introduction

Prostate cancer (PCa) is one of the most prevalent tumors of the urinary system, posing a significant health threat to men worldwide, particularly in developing countries [1]. PCa is the most common non‐cutaneous malignancy among men worldwide, with approximately 1.6 million new cases and 366,000 deaths reported annually [2]. According to estimates, 8937 new cases of PCa were reported in 2020, ranking it as the third most common malignancy among Iranian men [3]. Among US men, PCa has been identified as the second most common non‐cutaneous cancer after lung cancer. It is also a leading cause of cancer‐related mortality among men of all races and ethnicities, including those of Hispanic descent [4]. Between 95% and 98% of elderly individuals identified with prostate diseases have been found to have benign prostatic hyperplasia (BPH), PCa, or both as the primary organ disease [5]. PCa is a multifactorial disease influenced by a complex interplay of genetic and environmental factors [6]. PCa strategies may potentially impact the quality of life due to the burden of morbidity and adverse effects associated with active treatments such as surgery, radiotherapy, chemotherapy, and androgen deprivation, which are known to affect the quality of life negatively [7].

There are known, non‐modifiable risk factors such as age, ethnicity, and genetics for PCa [8]. Given the inability to modify non‐modifiable risk factors, opportunities remain for implementing policies that can mitigate the impact of modifiable risk factors (diet, physical activity, obesity, and lifestyle) through early identification and intervention. Prevention strategies for modifiable risk factors may vary across countries and should be communicated to patients and at‐risk individuals as soon as possible. Individuals at risk due to non‐modifiable factors such as family history, race, and age should receive counseling. Therefore, investigating all of these risk factors is essential for a better understanding of the etiology and onset of PCa.

Over the past decade, artificial intelligence (AI) has been increasingly integrated into medical practice, particularly in urology, where it is being explored and implemented for the diagnosis and treatment of PCa. AI techniques have the potential to rapidly analyze large volumes of data, such as medical images and tissue samples, thereby enhancing the accuracy of PCa diagnosis and prognosis [9]. In the healthcare domain, the digitization and storage of vast amounts of medical data have played a pivotal role in facilitating the application of AI‐based methods in disease diagnosis, treatment, and prediction [10]. The growing complexity and volume of healthcare data clearly highlight why AI techniques will be increasingly utilized across nearly all fields of medicine in the coming years [11].

Machines have become increasingly effective in discerning hidden patterns or insights from a given dataset through advancements in AI technologies, machine learning algorithms, and deep learning models grounded in mathematical principles and conditional statistical assumptions. These sophisticated algorithms have empowered AI‐based systems to enhance their predictive capabilities without explicit programming [12].

Machine learning (ML) is a subset of AI where algorithms learn from data without being explicitly programmed. ML is classified into supervised, semi‐supervised, and unsupervised categories [13]. Within supervised ML algorithms, input data comprises labeled data, and the model adjusts weights to achieve optimal fitting. Conversely, unsupervised learning involves unlabeled data, where algorithms identify hidden patterns in the dataset using clustering methods. The semi‐supervised approach combines principles from both supervised and unsupervised learning. The algorithm starts training on a small, labeled dataset and then uses this training to extract features from a larger, unlabeled dataset. In addition to these three categories, weakly supervised ML represents another class in the field of ML. ML techniques are increasingly routinely employed in the health sector to uncover hidden knowledge from massive datasets.

Numerous studies have widely employed ML algorithms to diagnose PCa and identify risk factors associated with PCa [14]. The effectiveness of ML‐based models for predicting the risk of PCa in the general population has been assessed in multiple research [15, 16].

Recent studies have shown that ML models, whether applied to imaging data or molecular sequences, can achieve high predictive accuracy and provide rapid, non‐invasive alternatives to traditional laboratory or biopsy‐based methods. These findings highlight the growing potential of advanced computational techniques in biomedical research and in the risk prediction of complex diseases such as PCa [17, 18]. These studies developed and validated ML‐based models for predicting the risk of PCa using huge datasets that included genetic, epigenetic, clinical, and imaging data [19, 20]. Outperforming conventional PSA‐based techniques, the results show that ML‐based models for PCa risk prediction have excellent accuracy, sensitivity, and specificity in identifying patients at high risk of developing PCa.

Since the risk factors related to PCa interact with each other, the present study was conducted to predict the risk of PCa and subsequently identify the influential factors contributing to its development using ML algorithms.

## 2 Materials and Methods

### 2.1 Study Design and Settings

To report this study, the ‘Transparent Reporting of a multivariable prediction model for individual prognosis or diagnosis (TRIPOD + AI)’ guideline was used [21]. This study was a retrospective study conducted in 2024 at Shahid Beheshti Urology Hospital, affiliated with Hamadan University of Medical Sciences, Iran. A total of 597 patients, with PCa and BPH, were included, selected from a pool of 1030 medical records. This study was conducted based on the following steps:

### 2.2 Feature Identification and Patient Selection

This stage involved identifying risk factors for PCa in patients diagnosed with it. Initially, the most significant risk factors were identified through an extensive literature review of the Web of Science, Scopus, Medline, and PubMed Central databases. Searching the databases was conducted from the beginning of 2000 to 2024. The keywords used for searching are listed in Table 1.

**Table 1:** Keywords used for searching databases.

Keywords
(“Prostatic Neoplasms” OR “Prostate Neoplasms” OR “Neoplasms, Prostate” OR “Neoplasm, Prostate” OR “Prostate Neoplasm” OR “Neoplasms, Prostatic” OR “Neoplasm, Prostatic” OR “Prostatic Neoplasm” OR “Prostate Cancer” OR “Cancer, Prostate” OR “Cancers, Prostate” OR “Prostate Cancers” OR “Cancer of the Prostate” OR “Prostatic Cancer” OR “Cancer, Prostatic” OR “Cancers, Prostatic” OR “Prostatic Cancers” OR “Cancer of Prostate”) AND (“Risk” OR “Risk Factors” OR “Risk Of Cancer” OR “Risk Factor” OR “Etiology” OR “causality” OR “risk prediction”)

A researcher‐developed questionnaire was subsequently utilized to validate the identified risk factors. The questionnaire comprised three sections: demographic information on specialists in urology, an assessment of the necessity of each risk factor by specialists, and an open‐ended question to introduce factors that the researcher did not consider. This questionnaire was designed to determine risk factors (patient demographics, lifestyle, medical history, laboratory tests, therapeutic classes, and diet) that can be effective in PCa.

A panel of eleven urology specialists assessed the questionnaire's content validity. Test‐retest reliability was also evaluated with a 10‐day interval. Finally, urologists validated the proposed risk factors through a two‐round Delphi survey. Experts were asked to review the initial list of parameters and rate each item based on its importance in PCa mortality on a 5‐point Likert scale, ranging from 1 (strongly disagree) to 5 (strongly agree).

After calculating the mean and standard deviation of the influencing factors, those with a mean score below 2.5 (indicating less than 50% agreement among specialist physicians) were excluded from the study. Factors with a mean score between 2.5 and 3.75 proceeded to the second round of the Delphi study, and finally, risk factors with a mean score of 3.75 or higher (indicating 75% agreement) were entered into the study. At this stage, we conducted data analysis using IBM SPSS Statistics version 16 (IBM Corporation, Armonk, NY, USA).

After performing a literature review coupled with a two‐round Delphi survey, based on the finalized feature set, the researcher subsequently extracted data from the medical records of patients who had undergone relevant examinations, including transrectal ultrasound (TRUS), and whose prostate biopsy pathology results were considered the ‘gold standard.’ Our study only included hospitalized patients diagnosed with a positive TRUS biopsy. Patients who were missing medical data or those admitted for other cancers were not included in the study. To investigate the dietary data that was not documented in the patient's medical records, telephone interviews using the Food Frequency Questionnaire (FFQ) [22], were conducted with the patients. Using the following portion sizes of typical Iranian food, the questionnaire recorded the frequency of food consumption of the participants: Participants were requested to indicate how often they consumed each food item, specifying the frequency on a daily, weekly, monthly, or yearly scale [23]. All collected data were subsequently entered into an Excel file. (Microsoft Corporation, United States).

### 2.3 Preprocessing

Records with more than 12 missing values and cases whose missing information could not be obtained through patient telephone interviews were excluded from the study. This approach ensured data quality and prevented the introduction of selection bias due to excessive data loss. Patient names were anonymized and added to an Excel sheet using numerical identifiers (1–597).

Missing data were managed using a two‐step imputation strategy. For quantitative variables, missing values were imputed using the K‐Nearest Neighbors (KNN) imputation algorithm, which estimates missing values based on the similarity of observations in multidimensional feature space. This method preserves the underlying distribution of numeric variables and reduces the risk of bias associated with single‐point substitution techniques [24].

For qualitative (categorical) variables, missing data were handled using mode imputation, where each missing entry was replaced with the most frequently occurring value in its respective variable.

All categorical variables were converted to numerical format using Label Encoding. Continuous predictors were then standardized using *z*‐score normalization (StandardScaler) to ensure uniform scaling across all models.

### 2.4 Model Development

Predictive classification models were developed using six ML algorithms selected through a comprehensive literature review [25]: three classical methods, specifically logistic regression (LR), gradient boosting (GB), and random forest (RF), and three modern advanced methods, specifically support vector machine (SVM), extreme gradient boosting (XGBoost), and neural network (multi‐layer perceptron).

LR, a statistical method for analyzing a dataset in which there is one binary dependent variable, was employed to examine the relationship between one or more independent variables and the outcome [26]. GB, an ensemble method, combines multiple weak learners to create a strong final model [27]. The RF algorithm helps minimize the risk of overfitting by combining the predictions of multiple decision trees, leading to improved accuracy. Additionally, it is capable of handling large datasets efficiently [28]. The method's main advantages are its ease of use, robustness against overfitting, and straightforward model interpretation. SVM with radial basis function (RBF) kernel was employed for its effectiveness in high‐dimensional spaces and non‐linear classification problems [29]. XGBoost, an optimized distributed GB library, was selected for its computational efficiency and state‐of‐the‐art performance on structured data [30]. Neural networks (multi‐layer perceptron) with hidden layers were implemented to capture complex nonlinear relationships in the data [31]

To ensure robust model evaluation, we implemented 10‐fold stratified cross‐validation in addition to the standard train‐test split. This approach provides a more reliable estimate of model performance and reduces the risk of overfitting [32].

Python 3.12 (Python Software Foundation), along with libraries such as Matplotlib, NumPy, Seaborn, Pandas, Scikit‐learn, XGBoost, and SHAP, was used for data analysis. To assess the model, an 80:20 split was employed for training and testing, respectively, complemented by 10‐fold stratified cross‐validation. A variety of performance evaluation pre‐specified metrics, including accuracy, precision, recall, F1‐score, sensitivity, specificity, area under the receiver operating characteristic curve (AUC‐ROC), and support, were employed to assess the performance of the prediction algorithm. Additionally, SHAP (SHapley Additive exPlanations) analysis was performed to provide model interpretability and identify the most influential features in predictions [33].

### 2.5 Ethical Considerations

Patient data were collected confidentially, with no identifying information recorded or shared outside the research team. The study followed the ethical guidelines of the Declaration of Helsinki, and informed consent was obtained from all participants. The study methodology was explained to patients, and all procedures complied with relevant guidelines and regulations.

Ethical approval (IR.UMSHA.REC.1403.042) was obtained from the Ethics Committee of Hamadan University of Medical Sciences. The Gemini AI tool (A Google product) was also used for language editing.

### 2.6 Statistical Analysis

For statistical analysis and comparison of the two groups of PCa and BPH, IBM SPSS Statistics version 16 (IBM Corporation, Armonk, NY, USA) was used. Quantitative variables are expressed as mean ± standard deviation (SD), whereas qualitative variables are presented as frequencies and percentages. To compare quantitative variables between the two groups, the independent t‐test was used [34]. The chi‐square test (χ^2^) was used for qualitative variables between the two groups. *P*‐value (probability value indicating statistical significance) < 0.05 was considered to indicate a statistically significant difference. All statistical analyses were performed using two‐sided hypothesis tests. Descriptive statistics presented in Table 4 constitute pre‐specified analyses, whereas exploratory analyses, including post‐hoc subgroup analyses and SHAP‐based feature interpretation, are reported separately in Figures 4 and 5.

## 3 Results

The research findings are the following, based on the research steps.

### 3.1 Step 1: Risk Factors Identification

The literature review results identified 69 risk factors. These 69 factors were then surveyed by experts in two rounds of the Delphi technique. Eleven experts participated in these two rounds of the Delphi technique, whose demographic information is given in Table 2.

**Table 2:** Physicians' demographics.

Variable	Subclass	Mean ± SD	Percentage of Frequency
Gender	Female	—	1 (9.1%)
	Male	—	10 (90.9%)
Education degree	Resident	—	4 (36.4%)
	Urologist	—	7 (63.6%)
Workplace	Operating room	—	11 (100%)
	Other units	—	—
Age	—	42.27 ± 12.32	—
Work experience (years)	—	13.82 ± 9.85	—

Abbreviation: SD, standard deviation.

Based on the experts' opinions, 20 factors were eliminated in these two rounds, and 49 factors remained, which were used as input to ML algorithms. These features were categorized into seven groups: patient demographics information, lifestyle, medical history, laboratory tests, diet, medication regimens, and other factors. These 49 factors are listed in Table 3.

**Table 3:** Predicting factors for the morbidity of prostate cancer in patients.

Classes	Number of suggested features	Delphi round			Final features	Included features	Excluded features
		< 50%	> 75%	50%–75%			
Demographic	7	2	3	2	5	Age, family history, material status, BMI, blood group	Income status, level of education
Life style	6	1	5	0	5	Smoking habit, Alcohol habit, Hookah, opium consumption, physical activity	Shiftwork
History of diseases	17	4	6	9	11	Type 2 diabetes, Blood pressure, TURP history, open prostatectomy history, BPH history, vasectomy, acute prostatitis, chronic prostatitis, UTI, epididymo‐orchitis, acute pyelonephritis	Breast cancer, schizophrenia, periodontal disease, Parkinson's disease, Inflammatory bowel disease, HPV
Laboratory tests	12	2	1	9	9	Hb, cr, urea, Na, AST, ALT, Plt, free PSA, total PSA	LDL, Triglyceride, Vitamin D
Diet	13	1	9	3	12	Tomato consumption Vegetable or fruit consumption, Fish consumption Red meat consumption, Soy consumption Beans consumption, Nuts consumption, Dairy consumption, Mushroom consumption, Green tea consumption, Coffee consumption, Egg consumption	Proteins diet
Therapeutic plans	10	4	4	2	6	Statins medications, BS medications, BP medications, ASA Sex hormones, steroids Zinc consumption	Salbutamol, warfarin, bismites, multivitamins
Other	4	2	1	1	1	Organophosphate	Exposure to Sun, Exposure cadmium, Baldness

Abbreviations: ALT, alanine aminotransferase; ASA, acetylsalicylic acid; AST, aminotransferase aspartate; BMI, body mass index; BP medications, blood pressure medications; BS medications, blood sugar medications; Cr, creatinine; Hb, hemoglobin; Plt, Plates; TURP, transurethral resection of the prostate; UTI, urinary tract infection.

### 3.2 Step 2: Data Collection

**Figure 1:**
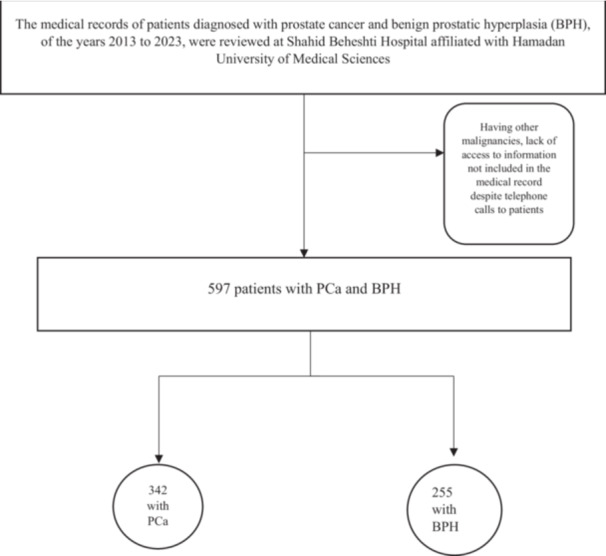
Flowchart illustrating the screening process of medical records for patients diagnosed with prostate cancer (PCa) and benign prostatic hyperplasia (BPH), and the final sample size included in the study.

At this stage, first, the medical records of patients with PCa and BPH were retrieved, and 1030 medical records were reviewed. Since several data factors were incomplete in the retrieved medical records, or some patients did not meet the inclusion and exclusion criteria, 597 medical records were included in the study out of 1030 (see Figure 1). In these 597 medical records, several data elements were also incomplete, and their information was obtained by contacting the patients. 341 patients (57.2%) had PCa, and 255 patients (42.8%) had BPH (Figure 1). The median age of the participants was 69.95 years (interquartile range 30–96) in the PCa group and 69.06 in the BPH group. The median body mass index (BMI) of participants was 25.88 kg/m^2^ (Table 4). Information about dietary factors was obtained using the FFQ questionnaire during telephone calls made by Serveh Mohammadi to patients. Participants' food intakes were assessed using a 160‐item semi‐quantitative FFQ [22] Table 4.

**Table 4:** General characteristics of the participants in the study.

	Features (quantitative)	Group name (*n* = PCa) mean ± SD	Group name (*n* = BPH) mean ± SD	*p*‐Value^a^
	Age (year)	69.95 ± 10.12	69.06 ± 30.63	0.10
	BMI (Kg/m^2^)	25.88 ± 2.64	25.88 ± 1.81	0.98
	Aspartate Aminotransferase	38.17 ± 65.28	23.51 ± 9.57	< 0.001
	Alanine aminotransferase	31.19 ± 87.37	23.30 ± 14.88	0.15
	creatinine	1.65 ± 1.38	1.29 ± 0.63	< 0.001
	Na	139.17 ± 3.86	57.18 ± 45.76	< 0.001
	Urea	57.18 ± 45.76	41.04 ± 16.56	< 0.001
	Free PSA	7.57 ± 11.09	3.58 ± 2.68	< 0.001
	Total PSA	41.59 ± 34.51	15.91 ± 14.14	< 0.001
	Hemoglobin	13.30 ± 16.74	14.31 ± 1.85	0.34
	Platelet count	21.30 ± 83.06	22.40 ± 11.11	0.07

#	Features (qualitative)	Frequencies		*p*‐Value^b^
		PCa	BPH	
Blood groups	A^+^	181 (60.74)	117 (39.26)	0.62
	A^−^	4 (40)	6 (60)	
	B^+^	54 (54.55)	45 (45.45)	
	B^−^	6 (54.55)	5 (45.45)	
	O^+^	72 (54.14)	61 (45.86)	
	O^−^	6 (66.67)	3 (33.33)	
	AB^+^	19 (51.35)	18 (48.65)	
	AB^−^	0 (0)	0 (0)	
Marital statues	Married	315 (55.65)	251 (44.35)	
	Single	23 (85.19)	23 (85.19)	
	Divorced	4 (100)	0 (0)	
Family history of cancer	Yes	139 (60.7)	90 (39.3)	0.13
	No	118 (58.71)	83 (41.29)	
Smoking status	Never smokers	247 (55.51)	198 (44.49)	0.32
	Current smokers	14 (60.87)	9 (39.13)	
	Former smokers	81 (62.79)	48 (37.21)	
Alcohol consumption	Former drinker	2 (100)	0 (0)	0.22
	Current drinker	0 (0)	0 (0)	
Hookah consumption	Never	332 (56.75)	253 (43.25)	0.07
	Former	10 (83.33)	2 (16.67)	
	Current	0 (0)	0 (0)	
Opium consumption	Never	240 (53.81)	206 (46.19)	0.01
	Former	99 (67.81)	47 (32.19)	
	Current	3 (60)	2 (40)	
Physical activity	Low	192 (56.64)	147 (43.36)	0.31
	Middle	146 (59.11)	101 (40.89)	
	High	4 (36.36)	7 (63.64)	
Type2 diabetes	Yes	40 (64.52)	22 (35.48)	0.22
	No	302 (56.45)	233 (43.55)	
Hypertension	Yes	101 (56.11)	79 (43.89)	0.70
	No	241 (57.79)	176 (42.21)	
History of BPH	Yes	51 (96.23)	2 (3.77)	< 0.001
	No	291 (53.49)	253 (46.51)	
Acute prostatitis	Yes	51 (96.23)	2 (3.77)	< 0.001
	No	341 (57.41)	253 (42.59)	
Chronic prostatitis	Yes	0 (0)	11 (100)	< 0.001
	No	342 (58.36)	244 (41.64)	
UTI	Yes	23 (71.88)	9 (28.13)	0.09
	No	319 (56.46)	246 (43.54)	
Epididymo‐orchitis	Yes	8 (100)	0 (0)	0.01
	No	334 (56.71)	255 (43.29)	
Acute pyelonephritis	Yes	22 (84.62)	4 (15.38)	
	No	320 (56.04)	251 (43.96)	
History of vasectomy	Yes	0 (0)	1 (100)	0.25
	No	342 (57.38)	254 (42.62)	
Statins medications	Yes	29 (63.04)	17 (36.96)	0.41
	No	313 (56.81)	238 (43.19)	
BS medications	Yes	41 (66.13)	21 (33.87)	0.14
	No	301 (56.26)	234 (43.74)	
BP medications	Yes	92 (55.09)	75 (44.91)	0.50
	No	250 (58.14)	180 (41.86)	
ASA	Yes	49 (59.04)	34 (40.96)	0.73
	No	293 (57)	221 (43)	
Exposure to organophosphate	Yes	252 (62.69)	150 (37.31)	< 0.001
	No	90 (46.15)	105 (53.85)	
Sex hormones, steroids	Yes	3 (100)	0 (0)	0.13
	No	339 (57.07)	255 (42.93)	
Zinc consumption	Yes	1 (100)	0 (0)	0.39
	No	341 (57.21)	255 (42.79)	
History of TURP	Yes	27 (96.43)	1 (3.57)	< 0.001
	No	315 (55.36)	254 (44.64)	
History of open prostatectomy	Yes	18 (90)	2 (10)	0.003
	No	324 (56.15)	253 (43.85)	
Tomato consumption	Negative	6(100)	*	< 0.001
	Twice a Year	*	*	
	Once every 2 months	*	*	
	Once a month	*	*	
	Twice a month	*	*	
	Once a Week	*	*	
	Twice a Week	*	*	
	Three times a Week	*	*	
	Four times a week	151(65.94)	*	
	Daily			
Vegetable or fruit consumption	*	*	*	0.04
Red meat consumption	*	*	*	< 0.001
Fish consumption	*	*	*	< 0.001
Soy consumption	*	*	*	0.009
Beans consumption	*	*	*	0.98
Nuts consumption	*	*	*	0.12
Dairy consumption	*	*	*	< 0.001
Mushroom consumption	*	*	*	< 0.001
Green tea consumption	*	*	*	0.24
Egg consumption	*	*	*	< 0.001
Coffee consumption	*	*	*	0.33

*Note:* Statistically significant as *p* < 0.05.

Abbreviations: ASA: acetylsalicylic acid; BMI: body mass index; BP medications: blood pressure medications; BS medications: blood sugar medications; PSA: prostate‐specific antigen; SD: standard deviation; TURP: transurethral resection of the prostate; UTI: urinary tract infection.

a Independent *t*‐test.

b Chi‐square test (*χ*²).

### 3.3 Step 3: Data Preprocessing

Human Immunodeficiency Virus (HIV) and Hepatitis C Virus (HCV) with missing values above 85% were excluded from the data collection. In total, according to specialists' opinions and after removing the factors with missing values, 49 factors were finally used to predict the morbidity of PCa. Also, in this step of the study, outliers and missing data were managed.

### 3.4 Step 4: Modeling With Machine Learning Algorithms and Evaluation

Six ML algorithms were used to predict PCa and identify risk factors, with data analyzed using Python 3.12 libraries. Evaluation metrics showed that XGBoost outperformed the others, with an accuracy of 77.50%, sensitivity of 74.51%, and specificity of 79.71%. LR had the second‐best performance (76.67%), while SVM had the lowest accuracy (Table 5).

**Table 5:** Comparative performance analysis of machine learning techniques.

	Accuracy	Sensitivity	Specificity	Precision	F1‐score	AUC‐ROC
XGBoost	0.7750	0.7451	0.7971	0.7308	0.7379	0.8289
Logistic regression	0.7667	0.6275	0.8696	0.7805	0.6957	0.8448
Gradient boosting	0.7667	0.7059	0.8116	0.7347	0.7200	0.8315
Random forest	0.7583	0.6078	0.8696	0.7750	0.6813	0.8305
Neural network	0.7500	0.6471	0.8261	0.7333	0.6875	0.8124
Support vector machine	0.7417	0.6667	0.7971	0.7083	0.6869	0.8440

Table 6 presents the detailed class‐wise performance metrics of all implemented ML algorithms. The XGBoost algorithm demonstrated superior overall performance, achieving the highest weighted average F1‐score of 0.78, followed closely by GB at 0.77.

**Table 6:** Performance metrics of machine learning algorithms across classes.

Metric	XGBoost	Logistic regression	Gradient boosting	Random forest	Neural network	Support vector machine
Class 0 (BPH)						
Precision	0.81	0.76	0.79	0.75	0.76	0.76
Recall	0.80	0.87	0.81	0.87	0.83	0.80
F1‐score	0.80	0.81	0.80	0.81	0.79	0.78
Support	69	69	69	69	69	69
Class 1 (cancer)						
Precision	0.73	0.78	0.73	0.78	0.73	0.71
Recall	0.75	0.63	0.71	0.61	0.65	0.67
F1‐score	0.74	0.70	0.72	0.68	0.69	0.69
Support	51	51	51	51	51	51
Macro Avg						
Precision	0.77	0.77	0.76	0.76	0.75	0.74
Recall	0.77	0.75	0.76	0.74	0.74	0.73
F1‐score	0.77	0.75	0.76	0.74	0.74	0.73
Support	120	120	120	120	120	120
Weighted Avg						
Precision	0.78	0.77	0.77	0.76	0.75	0.74
Recall	0.78	0.77	0.77	0.76	0.75	0.74
F1‐score	0.78	0.76	0.77	0.75	0.75	0.74
support	120	120	120	120	120	120

Abbreviation: BPH, benign prostatic hyperplasia.

**Figure 2:**
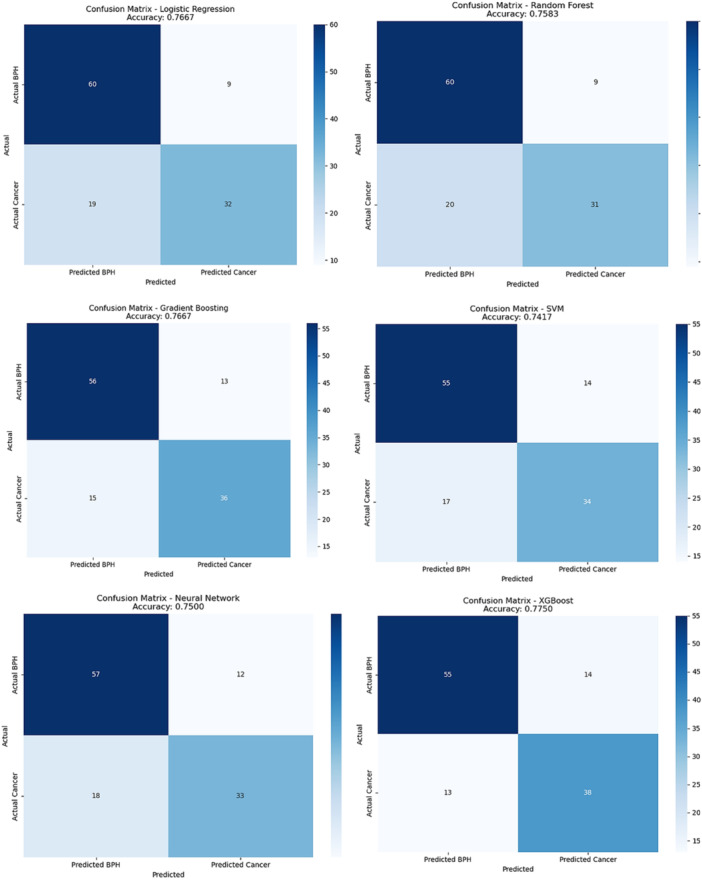
Comparison of confusion matrices of machine learning models for BPH and cancer classification.

In Figure 2, the confusion matrices for various ML models, including LR, RF, GB, SVM, Neural Network, and XGBoost, are presented. These matrices illustrate the performance of each model in distinguishing between the two classes, BPH (benign) and Cancer (malignant). Among the evaluated models, XGBoost achieved one of the highest accuracies (0.7750) and demonstrated strong performance in correctly identifying cancer cases.

**Figure 3:**
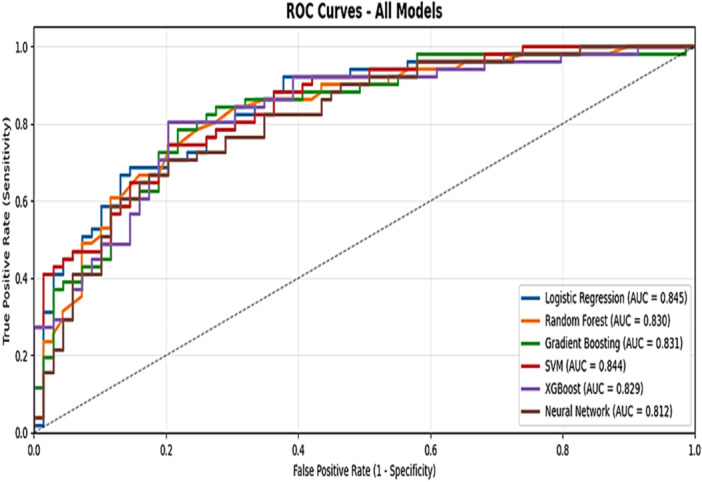
Receiver Operating Characteristic (ROC) curves comparing the performance of six classification models for prostate cancer prediction.

In Figure 3, the ROC curves of various ML models are presented to compare their ability to distinguish between the two classes, BPH and Cancer. The horizontal axis represents the False Positive Rate, while the vertical axis indicates the True Positive Rate. Based on the AUC (Area Under the Curve) values, which serve as an indicator of model quality, it can be observed that the LR and SVM models, with AUCs of 0.845 and 0.844, respectively, achieved the best performance among the evaluated models. These two models were able to identify cancer cases with higher accuracy and demonstrated better discrimination capability compared to the other models.

**Figure 4:**
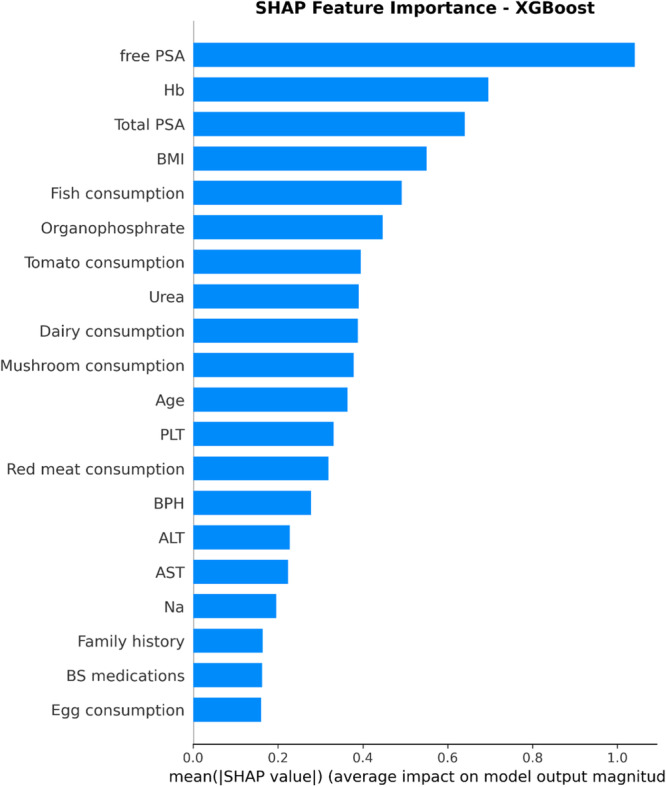
Feature importance ranking based on SHAP values for XGBoost model. ALT, alanine aminotransferase; AST, aspartate aminotransferase; BMI, body mass index; BPH, benign prostate hyperplasia; Hb, hemoglobin; PLT, platelet; PSA, prostate‐specific antigen.

The analysis of feature importance using SHAP values in the XGBoost predictive model showed that the contributions of variables to the model's final prediction were not equal. Based on the mean absolute SHAP value, biochemical factors had the greatest influence on the model's predictions. Among these, free PSA was identified as the most important variable, followed by Hemoglobin (Hb) and total PSA, which had a very significant impact on the model's output. In contrast, lifestyle and dietary factors such as fish consumption and BMI ranked lower in importance (Figure 4).

**Figure 5:**
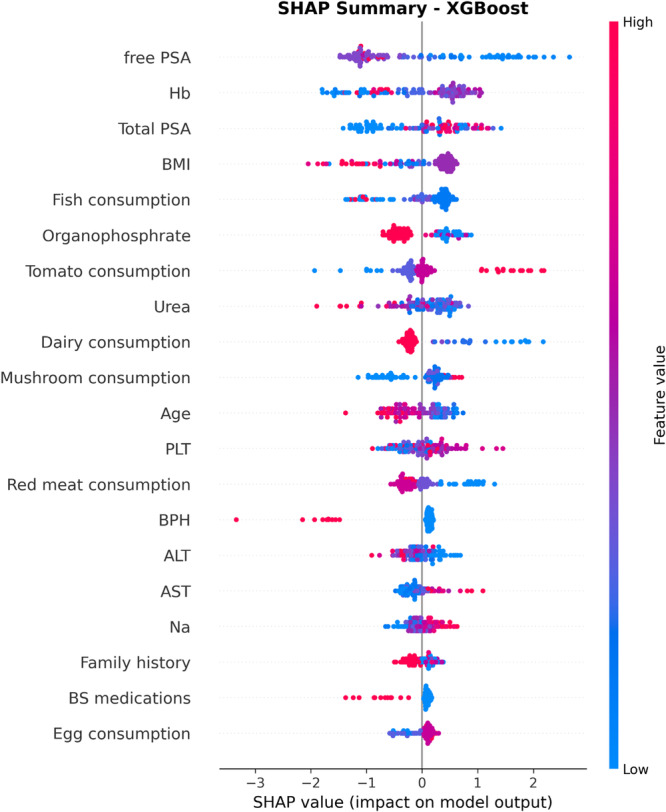
SHAP summary plot showing the impact of each feature on prostate cancer prediction. Color indicates feature value (red = high, blue = low); SHAP values reflect each feature's contribution to the model output.

In Figure 5, the SHAP summary plot for the XGBoost model is presented. This plot illustrates the importance and impact of each input variable on the model's output. The SHAP value represents the contribution of each feature in increasing or decreasing the probability of predicting cancer compared to BPH. Red points indicate high feature values, while blue points represent low values. The wider the dispersion of points for a given feature along the horizontal axis, the greater the influence of that feature in the model's decision‐making process.

According to this plot, the features Free PSA, Hb, and Total PSA have the greatest impact on the model's predictions; higher values of Free PSA and Total PSA substantially increase the likelihood of cancer. In contrast, other factors such as BMI, fish consumption, dairy consumption, and age play a smaller role in the classification task and exhibit more limited effects.

## 4 Discussion

This study used ML algorithms to investigate key risk factors for PCa based on medical records and phone calls from patients, divided into PCa and BPH groups. Recent investigations using donor‐derived biological samples and advanced deep‐learning methods have provided new insights into the genomic and histopathological characteristics of PCa, suggesting that molecular and image‐based approaches can offer valuable complementary information alongside conventional clinical risk factors [35, 36]. A total of 49 factors were analyzed, with free and total PSA levels and hemoglobin being the most significant laboratory‐related factors. In general, the results of the study showed that low levels of free PSA with high levels of total PSA increase the prediction of the model. Also, in demographic information, the high BMI factor was more influential in the prediction of the model for developing cancer. Also, among the diet‐related factors, increased fish consumption had a protective effect in our study.

PSA is a biomarker commonly used for the diagnosis of PCa, aiding in identifying patients who may require diagnostic evaluation [37]. PSA testing is typically performed for one of two purposes: investigating patients presenting to general practitioners (GPs) or primary care physicians with lower urinary tract symptoms (LUTS) [38], or screening patients who seek evaluation for PCa [39]. Patients with elevated PSA levels should undergo PCa evaluation via magnetic resonance imaging (MRI) or prostate biopsy using the TRUS method [40]. However, the diagnostic accuracy of PSA in patients with LUTS remains questionable. PSA testing is conducted on peripheral blood samples and reported in nanograms per milliliter (ng/mL) [39]. In our study, PSA levels were analyzed in two groups: patients with PCa and those with BPH. The aim was to evaluate the sensitivity of PSA as a key parameter for PCa, with both total and free PSA levels being higher than other factors. Nevertheless, a systematic review by Merriel and colleagues concluded that PSA is highly sensitive but not specific for diagnosing PCa in symptomatic patients [39]. Our findings further demonstrated that among laboratory parameters, hemoglobin levels exhibited greater variability between the PCa and BPH groups than other laboratory markers. This variability suggests hemoglobin is a potential diagnostic factor for PCa. Wang and colleagues also concluded in their study that a model's ability to predict PCa based on features such as laboratory tests, including hemoglobin, showed high accuracy°and°consistency [41].

In our study, we concluded that high BMI is associated with an increased risk of PCa. A recent systematic review and meta‐analysis also concluded that high BMI is associated with an increased risk of PCa, which is consistent with the results of our study [42]. As one study concluded, Obesity causes chronic inflammation in fat tissue, leading to oxidative stress, inflammatory cytokines, and high insulin/IGF levels. These factors promote Epithelial to Mesenchymal Transformation (EMT), dramatically increasing prostate cancer's aggressiveness and metastatic potential [43].

One of the most modifiable risk factors for PCa is diet. In our study, fish consumption in our study had a possible protective effect against cancer. A recent systematic review and meta‐analysis concluded that overall, the consumption of fish and omega‐3 fatty acids does not appear to significantly influence the incidence of PCa, but it is associated with a reduction in mortality from the disease. This discrepancy between incidence and mortality may be attributed to the protective role of omega‐3s in enhancing the response of tumors to ablation therapy. Evidence suggests that these fatty acids can increase the omega‐3 content within prostate tumors, improve treatment response, and slow progression to androgen‐independent growth. Additionally, their anti‐inflammatory and cholesterol‐lowering effects may suppress tumor growth and angiogenesis, ultimately lowering the risk of death. The inconsistent findings in some studies regarding fish consumption and PCa may be due to variations in fish types (dark meat fish, white fish, lean fish, Shrimp, and scallops), preparation methods, and the actual amount consumed [44]. Dairy product consumption (milk, yogurt, cheese) and red meat intake were associated with increased PCa incidence, consistent with previous studies [45]. Animal studies have shown that compounds such as heterocyclic amines (HCAs), polycyclic aromatic hydrocarbons (PAHs), and N‐nitroso compounds (NOCs), produced during high‐temperature cooking of meat and meat products, are carcinogenic [46].

A high ratio of saturated fatty acids has been associated with various chronic diseases, including cancer, according to previous studies. One study reported no association between overall dairy consumption and PCa risk, which contradicts our findings [47]. Nonetheless, factors such as fat percentage and fermented dairy products might yield differing results [48].

In this study, a history of BPH was associated with a higher likelihood of PCa compared with other medical conditions. BPH, a non‐cancerous enlargement of the prostate, occurs through cellular proliferation [49]. Anatomically, the prostate is divided into three primary zones based on embryological origin and unique function: the transitional zone (TZ), the peripheral zone (PZ), and the central zone [50]. While many clinicians consider TZ the primary site for BPH development and PZ the region most associated with PCa, regional anatomy studies are crucial for better understanding BPH and PCa interactions [51].

However, whether BPH serves as a premalignant lesion remains unclear [15]. Despite shared features such as hormonal dependence and pharmacological responses to hormonal antagonists, a large epidemiological study is needed to define the precise relationship between these conditions [52].

In our study, six ML algorithms (XGBoost, LR, GB, RF, Neural Network (Multi‐Layer Perceptron), SVM) were developed and evaluated. Among these, the XG Boost model showed the best overall performance, with an accuracy of 77.50%, sensitivity of 74.51%, and a weighted average F1‐score of 0.78. LR achieved a slightly higher AUC‑ROC of 0.8448; its lower sensitivity (0.6275) limits its utility when the clinical priority is to minimize missed cancer diagnoses [53]. Other models, such as RF and GB, performed worse than XGBoost. They had lower sensitivity and an F1 score in the PCa class. Also, SVM and MLP generally performed worse in our dataset.

In the study by Arafa et al., who evaluated ML models based on prostate biopsies, they showed that XGBoost and RF have high accuracy in cancer detection, which is consistent with the results of our study [54]. Also Zhou et al. They concluded that the XGBoost model developed the highest accuracy compared to other ML methods (decision tree learning, lasso, MLP, and SVM) in distinguishing between PCa and BPH using eight noninvasive predictor variables, which is consistent with the results of our study in identifying the best model [55].

Key strengths of this study include the use of ML for improved accuracy and detailed variable analysis, the application of the Delphi method ensured systematic expert input for feature selection, enhancing the robustness of the model, and a multidimensional evaluation of PCa risk factors, including demographics, lifestyle, medical history, laboratory findings, and dietary data. This comprehensive approach offers valuable insights into a population sharing similar geographic and ethnic characteristics.

This study has several limitations:

Single‐center study: The study was conducted in a single hospital, which may limit the generalizability of the results, although the center covered patients from several cities and regions. Data limitations: Of 1,030 medical records, only 597 were included in the analysis. Records with excessive missing data were excluded to ensure data quality, while missing information in some cases was supplemented through telephone interviews. To further reduce potential bias, data preprocessing techniques, including KNN imputation and 10‐fold cross‐validation, were applied.

Recall bias in dietary data: Dietary information collected retrospectively with a telephone FFQ may be subject to measurement error and recall bias, although the use of a standard FFQ and cross‐checking with medical records has partially reduced this limitation.

Feature subjective bias: Although feature selection was conducted using expert consensus through the Delphi method, some degree of subjectivity may remain, which could influence the prioritization of factors.

Possibility of circularity: Although PSA was included due to its widespread use in clinical screening and relevance to real‐world settings, we acknowledge that its inclusion may introduce circularity. Future studies should consider excluding PSA to identify independent risk factors more accurately.

## 5 Conclusion

This study found that key factors—such as total and free PSA levels and serum hemoglobin—play a crucial role in improving the performance of ML models for predicting PCa. Among the algorithms tested, XGBoost delivered the most accurate results, suggesting its potential as a valuable tool to support clinical decision‐making. While the findings highlight a promising use of ML in oncology, they also highlight the need for further research using larger and more diverse patient populations to validate and refine these predictive models for broader clinical applications.

## Author Contributions

**Serveh Mohammadi:** visualization, project administration, formal analysis, writing, and revising. **Behzad Imani:** writing draft and editing, supervision, investigation, methodology, and funding acquisition. **Soheila Saeedi:** validation, software, formal analysis, writing, and revising. **Mohammad Ali Amirzargar:** writing original draft, investigation, data curation.

## Disclosure

All authors have read and approved the final version of the manuscript. Behzad Imani had full access to all of the data in this study and takes complete responsibility for the integrity of the data and the accuracy of the data analysis.

## Conflicts of Interest

The authors declare no conflicts of interest.

## 1 Transparency Statement

The lead author Behzad Imani affirms that this manuscript is an honest, accurate, and transparent account of the study being reported; that no important aspects of the study have been omitted; and that any discrepancies from the study as planned (and, if relevant, registered) have been explained.

## Data Availability

The authors confirm that the data supporting the findings of this study are available within the article [and/or] its supporting materials.
